# Supplementary material for: The facultative intracellular symbiont Lariskella is neutral for lifetime fitness and spreads through cytoplasmic incompatibility in the leaffooted bug, Leptoglossus zonatus
Source: Front Microbiol. 2025 Jul 10;16:1595917. doi: 10.3389/fmicb.2025.1595917 (PMC12288687; doi:10.3389/fmicb.2025.1595917)
Supplement: Supplementary file 1 [file Supplementary_file_1.docx]

Supplementary Material

# Supplementary Data

- Edwin analysis.R
- Edwin_phyloseq.Rdata
- RGLE data processing – Edwin.R

# Supplementary Figures and Tables

## Supplementary Figures

**
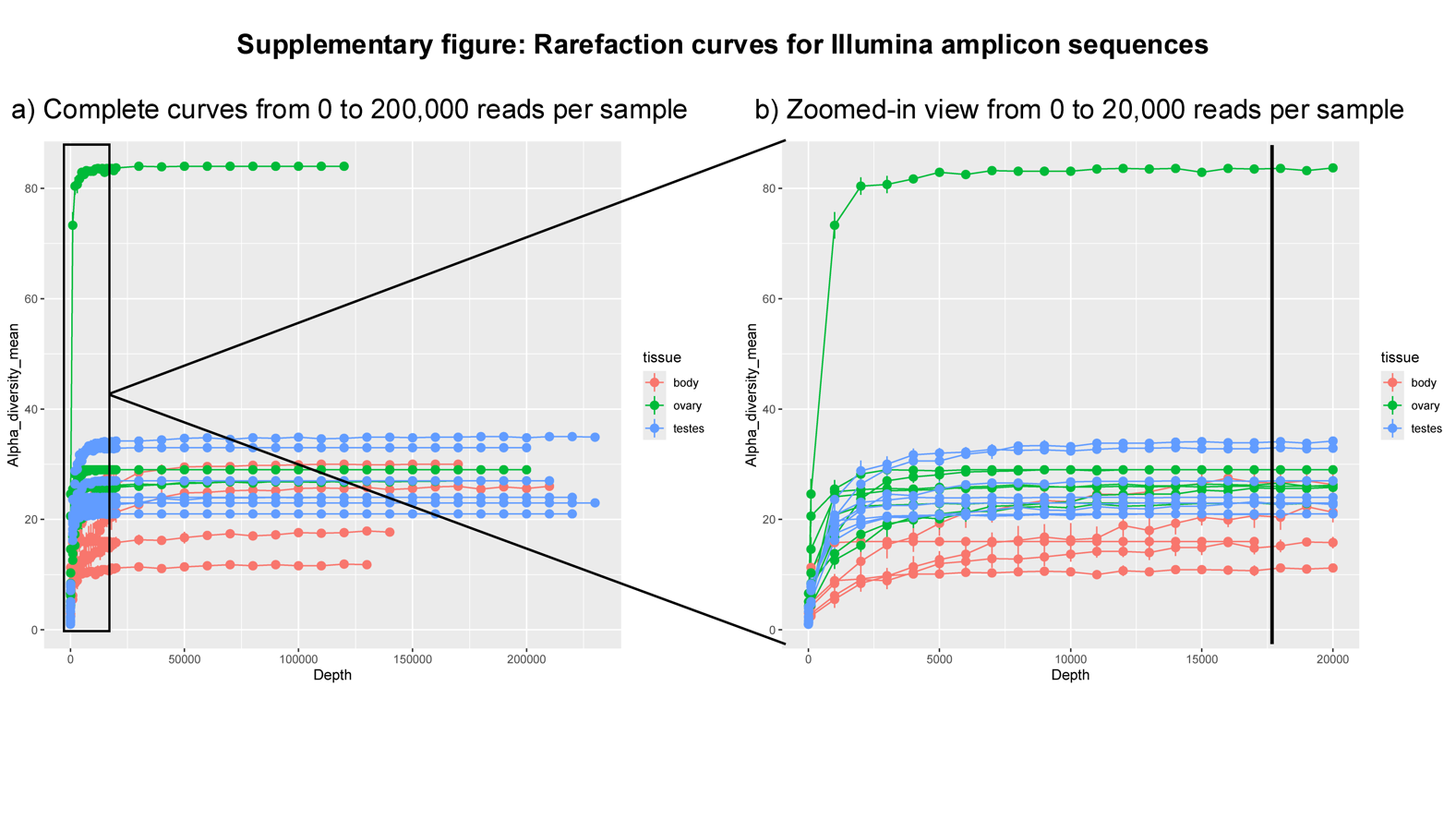
**

**Supplementary Figure 1.** Rarefaction curves showing alpha diversity of *Leptoglossus* zonatus whole-body, ovaries, and testes samples. Visual inspection of these curves indicate that bacterial community diversity was adequately characterized at the chosen rarefaction depth of 17, 875 reads per samples (vertical line in panel b), which was the lowest number of per samples reads in this dataset.
